# Inflammatory Markers in Cerebrospinal Fluid from Patients with Hydrocephalus: A Systematic Literature Review

**DOI:** 10.1155/2021/8834822

**Published:** 2021-02-02

**Authors:** Sara Diana Lolansen, Nina Rostgaard, Eva Kjer Oernbo, Marianne Juhler, Anja Hviid Simonsen, Nanna MacAulay

**Affiliations:** ^1^Department of Neuroscience, University of Copenhagen, Copenhagen, Denmark; ^2^Department of Neurosurgery, Rigshospitalet, Copenhagen, Denmark; ^3^Danish Dementia Research Centre, Department of Neurology, Rigshospitalet, Copenhagen, Denmark

## Abstract

**Objective:**

The aim of this systematic review was to evaluate existing literature on inflammatory markers in CSF from patients with hydrocephalus and identify potential markers capable of promoting hydrocephalus development and progression.

**Methods:**

Relevant studies published before December 3^rd^ 2020 were identified from PubMed, Embase, and reference lists. Studies were screened for eligibility using the predefined inclusion and exclusion criteria. Data from eligible studies were extracted, and sources of bias were evaluated. We included articles written in English investigating inflammatory markers in CSF from patients with hydrocephalus and control subjects. The review was conducted according to the PRISMA guidelines by three independent reviewers.

**Results:**

Twenty-two studies analyzed CSF from 311 patients with idiopathic normal pressure hydrocephalus (iNPH), 178 with posthemorrhagic hydrocephalus (PHH), 151 with other hydrocephalus diagnoses, and 394 control subjects. Fifty-eight inflammatory markers were investigated. The CSF of iNPH patients had increased CSF levels of IL-6, IL-1*β*, and LRG compared with control subjects, whereas the CSF of PHH patients had increased levels of IL-6, IL-18, and VEGF. CSF from patients with “other hydrocephalus diagnoses” had elevated IFN-*γ* compared to control subjects, and VEGF was increased in congenital hydrocephalus, spina bifida, and hydrocephalus associated with tuberculous meningitis compared with controls.

**Conclusion:**

IL-6, IL-1*β*, LRG, IL-18, VEGF, and IFN-*γ* are elevated in CSF from patients with hydrocephalus and may be involved in promotion of hydrocephalus development and progression. They may serve as novel disease biomarkers, and their signaling pathways may represent targets for pharmacological management of hydrocephalus.

## 1. Introduction

Hydrocephalus is characterized by excessive amounts of cerebrospinal fluid (CSF) accumulating within the brain ventricles resulting in an increased intracranial pressure [1]. The CSF accumulation can have detrimental consequences and results in severe cognitive and neurological impairments and ultimately death if untreated [2]. Hydrocephalus occurs in all age groups, but onset is the most frequent in infants and elderly, i.e., in age groups where the brain is the most vulnerable [3]. Although the term “hydrocephalus” applies to all, the clinical presentations, radiological features, and etiologies greatly vary [2]. In infants, hydrocephalus is most often caused by undiagnosed intrauterine conditions, congenital malformations, perinatal/infantile infections, or secondary intraventricular hemorrhage (IVH) due to germinal matrix bleeding [4–6]. Posthemorrhagic hydrocephalus (PHH) occurs in infants as well as older children and adults often as results of head trauma, IVH, or ruptured aneurysms (subarachnoid hemorrhage, SAH) [6]. In elderly, hydrocephalus often presents as idiopathic normal pressure hydrocephalus (iNPH), a condition without known etiology characterized by no or only slight increase in intracranial pressure, gait disturbances, urinary incontinence, and dementia. Although the etiologies vary tremendously in different types of hydrocephalus, emerging evidence indicates that neuroinflammation contributes to the pathogenesis [7]. Elevated levels of inflammatory cytokines have been found in serum and CSF from patients with hydrocephalus [8–10], and the brain tissue from patients with hydrocephalus shows signs of inflammation such as macrophage and microglial activation [11, 12]. In addition, anti-inflammatory drugs can reduce the incidence of hydrocephalus in humans and attenuate ventriculomegaly in animals [13, 14]. Activation of inflammatory pathways in the CSF-producing tissue, the choroid plexus, can promote CSF hypersecretion sufficient to cause ventriculomegaly in animals [15]. Moreover, prolonged inflammation can contribute to scarring and fibrosis [16, 17], which is proposed to disrupt CSF flow and impair CSF reabsorption [7, 18]. Inflammation may thus promote the development and progression of hydrocephalus via CSF hypersecretion and/or impaired CSF drainage. To identify potential inflammatory markers involved in the pathogenesis of hydrocephalus, we systematically reviewed the existing literature on inflammatory markers in CSF from patients with hydrocephalus. Hereby, we identified promising inflammatory markers for future experimental research that aims to elucidate whether inflammatory markers can alter CSF dynamics and contribute to hydrocephalus development and progression. If inflammatory markers are involved in the pathogenesis of hydrocephalus, they may serve as novel disease biomarkers, and their signaling pathways may represent potential targets for pharmacological management of hydrocephalus. There is a great need for new potential biomarkers in diagnostic and prognostic settings within the field of hydrocephalic conditions.

## 2. Methods

The systematic literature review was conducted in accordance with the Preferred Reporting Items for Systematic Reviews and Meta-Analyses (PRISMA) statement [19], but is not registered in PROSPERO (as it does not include clinical trials).

### 2.1. Eligibility Criteria

**Figure 1:**
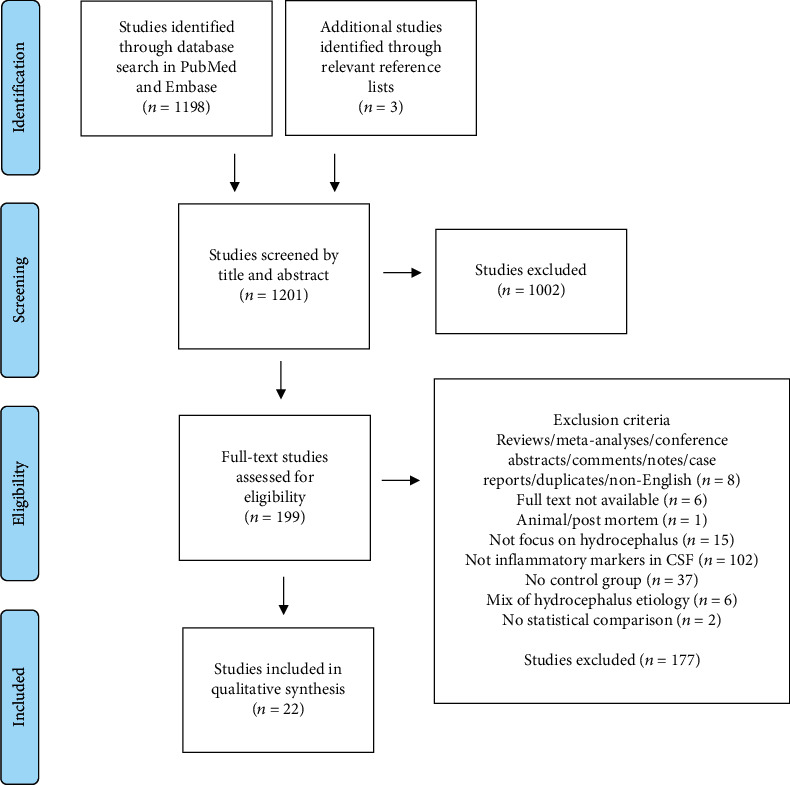
PRISMA flow diagram of the literature search strategy. The database search identified 1198 studies, and 3 additional studies were identified from reference lists. Screening by title and abstract led to the exclusion of 1002 studies. Full-text review for eligibility of the remaining 199 studies resulted in a final inclusion of 22 studies for the review.

Studies were included in the review if they were (1) original research, (2) written in English, and (3) available in full text. Studies were excluded from the review if they (1) were reviews, meta-analyses, conference abstracts, comments, notes (i.e. gray literature), case reports, duplicates, or not published in English; (2) were not available in full text; (3) were animal or postmortem studies; (4) did not focus on patients with hydrocephalus; (5) did not analyze CSF for presence of inflammatory markers; (6) did not include a control group; (7) did not separate patients with hydrocephalus of different etiologies into separate groups; or (8) did not make a statistical comparison between patients with hydrocephalus and control subjects (Figure 1).

### 2.2. Search Strategy

PubMed and Embase were searched for relevant studies published before December 3^rd^ 2020. The following search terms were applied in all four searches: “hydrocephalus” AND “cerebrospinal fluid” OR “CSF” OR “cerebrospinal fluid” AND “inflammation” OR “inflammatory” OR “inflammatory marker” OR “biomarker” OR “marker” OR “cytokine.” A unique search term was, in addition, applied to each of the four searches.

*Search 1* “normal pressure hydrocephalus” OR “NPH” OR “idiopathic normal pressure hydrocephalus” OR “iNPH.” The search was limited to studies published after January 1^st^ 2005 where the international iNPH guidelines were first presented and later published [20].

*Search 2* “hemorrhage” OR “bleeding” OR “posthemorrhagic” OR “post-hemorrhagic” OR “post hemorrhagic.”

*Search 3* “trauma” OR “traumatic” OR “post-traumatic” OR “posttraumatic” OR “post traumatic” OR “post head injury.”

*Search 4* “meningitis” OR “infection” OR “infectious” OR “bacterial” OR “viral” OR “encephalitis.” Additional relevant studies were identified and manually curated from reference lists.

### 2.3. Data Items

The following data were extracted from each study: first author's name, publication year, study design, patient groups, number of patients in each group, diagnostic criteria including inclusion and exclusion criteria, age, sex, type of medication given, type of surgery performed, CSF source, preanalytical CSF handling, analytical method, CSF marker(s) analyzed, and result of CSF analysis.

### 2.4. Data Extraction and Quality Assessment

Authors SDL, NR, MJ, AS, and NM designed the protocol and search strategy.

Initially, authors SDL and NR independently screened the studies by title and abstract. Studies that clearly violated the eligibility criteria were excluded. Afterwards, SDL and NR independently assessed the studies for eligibility by full-text review. After the independent assessment, SDL and NR collectively selected the studies for the final analysis. Authors SDL and EKO extracted data from relevant studies independently. After independent data collection, SDL and EKO collectively screened the extracted data, discussed any disagreements, and corrected any inconsistencies.

### 2.5. Synthesis of Results

Inflammatory markers were separated into two categories based on the degree of consistency in results among studies. Inflammatory markers were categorized as having *most evidence* when two or more studies reported consistent results, and a maximum of one study reported a contradictory result. Inflammatory markers were categorized as having *limited evidence* when results were reported from a single study.

### 2.6. Risk of Bias

The potential risk of bias was assessed by the evaluation of the following data from each study: study design, sample size, age and sex, diagnostic criteria, inclusion and exclusion criteria, medication, circumstances of CSF sampling, pre-analytical CSF handling, selection of inflammatory markers for CSF analysis, and analytical methods.

## 3. Results

### 3.1. Study Selection

The PubMed and Embase search identified 1198 potential studies for the review, and 3 additional studies were identified from relevant reference lists. The initial search thus revealed 1201 potential studies for the review. Screening by title and abstract resulted in an exclusion of 1002 studies. Full-text review for eligibility of the remaining 199 studies led to the exclusion of 177 further studies. Hence, 22 studies were included in the final analysis (Figure 1).

### 3.2. Study Characteristics

The 22 studies included in the review were cross-sectional, case-control studies. Five studies were prospective [21–25], while two were retrospective [26, 27]. The remaining 15 studies did not report whether a prospective or retrospective strategy was performed. The 22 studies analyzed CSF from patients with hydrocephalus, which were sorted into three categories reflecting different hydrocephalic disorders: iNPH patients (*N* = 311), PHH patients (*N* = 178), and patients with other hydrocephalus diagnoses (*N* = 151). The latter patient group included hydrocephalus associated with tuberculous meningitis (*N* = 44), spina bifida (*N* = 28), aqueduct stenosis (*N* = 25), acute hydrocephalus of unknown origin (*N* = 12), congenital hydrocephalus (*N* = 9), hydrocephalus following embolization of unruptured intracranial aneurysms (*N* = 8), fetal onset hydrocephalus (*N* = 7), late onset hydrocephalus (*N* = 7), arrested hydrocephalus (*N* = 6), and hydrocephalus with brain atrophy (*N* = 5). The CSF from patients with hydrocephalus was compared to CSF from control subjects (*N* = 394). The 22 studies investigated the presence of 58 inflammatory markers in total.

### 3.3. Analytical Methods

The 22 studies applied different analytical methods to detect presence of inflammatory markers in the CSF samples. The analytical methods included enzyme-linked immunosorbent assays (ELISAs) [21, 23–35], multiplex immunoassays [35–38], radioimmunoassays [32], electrochemiluminescence assays [39], a milliplex immunoassay [22], a cytokine antibody microarray kit [40], a quantitative sandwich enzyme immunoassay [41], and a bead-based human inflammatory cytokine kit [42]. Only three studies reported blinding of laboratory staff [21, 28, 36].

### 3.4. Inflammatory Markers in iNPH

CSF from iNPH patients was screened for presence of 36 inflammatory markers spread across 11 studies [25–27, 32–34, 36–39, 42]. Detailed information from each study is provided in supplementary Table 1. The inflammatory markers were sorted according to the evidence criteria, and the results are presented in Table 1.

IL-6, IL-1*β* , and LRG showed most evidence of increased levels in CSF from iNPH patients compared to control subjects (Table 1) [25, 33, 37, 38, 42], while SP-G and T*β*R-II showed limited evidence of increased levels. TIMP-4 showed limited evidence of decreased levels in CSF from iNPH patients compared to control subjects. The remaining inflammatory markers showed no significant differences in CSF levels between iNPH patients and control subjects, were reported with contradictory results, or were below the detection limit (Table 1).

### 3.5. Inflammatory Markers in PHH

CSF from PHH patients was screened for presence of 37 inflammatory markers spread across 12 studies [21, 23, 24, 26, 28–31, 34, 35, 40, 41]. Detailed information is provided in supplementary Table 2. The studies analyzed CSF from infants as well as adults and the pathological events preceding hydrocephalus thus differ between the included studies. However, due to the limited number of studies with adult PHH patients and the absence of any obvious differences in inflammatory CSF profiles, the results were not separated according to age. The inflammatory markers were sorted according to the evidence criteria and the results are presented in Table 2.

IL-6, IL-18, and VEGF showed most evidence of increased levels in CSF from PHH patients compared to control subjects [21, 26, 29, 30, 35, 40].

Sixteen inflammatory markers, CCL-3/MIP-1*α*, CCL-19, CXCL-10/IP-10, HGF, HMGB1, IL-1*α*, IL-4, IL-12, L1CAM, MMP-9, FasR, sFas, SP-G, sRAGE, TGF-*β*2, and TIMP-1, showed limited evidence of significantly increased levels in CSF from PHH patients compared to control subjects. Two inflammatory markers, TIMP-4 and XCL-1, showed limited evidence of significantly decreased levels in CSF from PHH patients compared to control subjects. The remaining inflammatory markers showed no significant differences in CSF levels between PHH patients and control subjects, were reported with contradictory results, or were below the detection limit.

### 3.6. Inflammatory Markers in Other Hydrocephalus Diagnoses

CSF from the mixed patient group with other hydrocephalus diagnoses was screened for presence of 30 inflammatory markers spread across seven studies [21, 22, 26, 30, 34, 40, 42]. As the patient groups and the studies included in this category are extremely heterogeneous, it is not possible to summarize data in a simple table. Detailed information is provided in supplementary Table 3. However, an interesting marker is IFN-*γ*, which showed most evidence of increased levels in CSF from patients with hydrocephalus compared to control subjects [22, 30].

Further, VEGF was found increased over that of control subjects in congenital hydrocephalus, spina bifida, and hydrocephalus associated with tuberculous meningitis [21, 22, 40].

### 3.7. Risk of Bias

Sources of bias that could potentially influence the findings presented in the review are assessed in the following.

#### 3.7.1. Sample Size

Ideally, the sample size should be large enough to detect differences that are clinically relevant [43]. However, practical issues such as costs and patient availability often limit the sample size [43]. The sample size of the patient groups included in the review ranged from 2 to 100 individuals. In some of the studies, the small sample size may have reduced the chance of detecting a significant difference due to low statistical power. A small sample size may thus explain some of the instances where levels of inflammatory markers were reported similar in patients with hydrocephalus and control patients. However, the systematic review was designed not to exclude studies based on the low sample size as such exclusion would itself bias the results and risk potential loss of information.

#### 3.7.2. Age and Sex

Studies are generally recommended to match for age and sex to control for confounding elements and increase study efficiency [44]. Of the 22 included studies, nine studies reported that patients were age-matched or similar in age [22, 27–32, 37, 38]. The age distribution was given in 10 of the remaining studies [21, 23, 25, 33–36, 39–41 and was not reported in three studies [24, 26, 42]. Of the 22 studies, four studies examined the correlation between age and levels of inflammatory markers in the CSF [23, 25, 32, 34]. Of these, only one study reported a significant correlation with age [25]. Of the three categories of patients with hydrocephalus, PHH patients exhibited the largest variation in the age distribution ranging from preterm infants [21, 23, 29–31, 35, 40, 41 to adults [24, 28, 34]. In addition to variations related to age-matching, findings presented in the PHH category may have been particularly prone to age variation across studies. Eleven of the 22 studies reported a sex distribution among the included patient groups [22, 25, 27, 32–36, 38–40] while it was not reported in the remaining studies [21, 23, 24, 26, 28–31, 37, 41, 42]. Only three studies reported that groups were sex-matched or had a similar sex distribution [22, 27, 28]. None of the 22 studies included in the review reported any effect of sex on the levels of inflammatory markers.

#### 3.7.3. Diagnostic Criteria

*(1) iNPH Patients*. In 2005, English international guidelines for the clinical diagnosis of iNPH were published [20]. Studies published prior to the publication of the international guidelines were excluded from the review to limit patient heterogeneity. Of the 11 studies investigating iNPH, two studies diagnosed patients according to the international guidelines from 2005 [27, 39]. Two studies [25, 33] referred to another set of iNPH guidelines, which were published in Japan in 2004 and translated to English in 2008 [45, 46]. Five studies did not refer to any guidelines but diagnosed patients based on imaging, presence of clinical symptoms, and physiological criteria [32, 36–38, 42]. The last two studies did not report how iNPH patients were diagnosed [26, 34].

*(2) PHH Patients*. Patients diagnosed with PHH are heterogeneous in relation to age of onset, underlying pathological event, and clinical presentation. The internationally accepted diagnostic criteria for PHH are yet to be established [6]. However, criteria and grading scales exist for the hemorrhagic events underlying PHH development (e.g., subarachnoidal hemorrhage; SAH, intraventricular hemorrhage; IVH). Of the 12 studies investigating PHH, six studies reported that patients developed PHH secondarily to IVH, and four studies reported the cause to be SAH, while one study reported PHH to develop from either SAH or IVH [21, 23, 24, 26, 28, 29, 31, 34, 35, 40, 41]. One study reported that patients developed PHH secondarily to intracranial hemorrhage but did not specify further [30]. Of the six IVH-related studies, different grading systems and criteria were used in three studies [21, 29, 31], while two of the four SAH-related studies reported use of a specific grading scale [24, 28].

*(3) Patients with Other Hydrocephalus Diagnoses*. Seven studies investigated patients with other hydrocephalus diagnoses [21, 22, 26, 30, 34, 40, 42]. The diagnoses were hydrocephalus associated with tuberculous meningitis, spina bifida or aqueduct stenosis, acute hydrocephalus of unknown origin, congenital hydrocephalus, hydrocephalus following embolization of unruptured intracranial aneurysms, fetal onset hydrocephalus, late onset hydrocephalus, arrested hydrocephalus, and hydrocephalus with brain atrophy. None of the studies reported use of specific guidelines.

#### 3.7.4. Inclusion and Exclusion Criteria

*(1) All Patients with Hydrocephalus*. The majority of the studies enrolled patients with hydrocephalus according to a certain diagnostic criteria. Only two studies specified the inclusion criteria that extended beyond the diagnostic criteria [32, 42]. One study included patients if they were free from other disorders of the central nervous system (CNS) and their mental status allowed neurophysiological tests to be performed [42]. The other study included patients if they were free from comorbidities with inflammatory or immunological diseases and had not received treatment with steroids or immunosuppressive drugs three months prior to the CSF sampling [32]. Four studies applied the exclusion criteria [23, 24, 27, 28]. The exclusion criteria were complications related to shunt surgery [27], history of CNS disease or active systemic disease [24, 28], presence of infections [23, 24], development of inflammatory reactions not associated with surgery [28], and exclusion of SAH patients below the age of 18 due to differences in CSF content, distinct SAH presentations, morphology of aneurysms, and outcome [24]. The absence of the specific inclusion and exclusion criteria in the majority of the studies suggests that the patient groups are heterogeneous. Hence, the results presented in this review may be compromised by patient heterogeneity. Only a few studies addressed presence of inflammation or other CNS disorders in the patient population [23, 24, 28, 32, 42].

*(2) Control Subjects*. The ideal control group should be the representative of the population from which patients arise [47]. Of the 22 studies, ten studies enrolled control patients who underwent workup for exclusion of sepsis or meningitis, or pathologies such as SAH, demyelinating disorders, and rhinorrhea [21, 23, 29–31, 34, 35, 40–42]. Five studies included control patients that underwent surgeries unrelated to hydrocephalus [22, 24, 26, 27, 38]. In addition, studies included control patients with noninflammatory conditions (headache, spinal disc herniation, dementia, and occipital neuralgia) [28, 32, 33, 37]. Two studies did not describe the control patients [25, 36]. One study reported that controls patients were healthy volunteers recruited from a population registry [39]. Of the 22 studies, 17 studies reported that control patients were free from neurological injuries, deficits, or disorders [21–25, 27–29, 31–33, 35, 38–42]. In addition to the studies where control subjects underwent workup for sepsis and meningitis, two studies reported absence of inflammation in control patients [22, 42]. Additionally, the specific exclusion criteria were reported in three studies [23, 28, 39]. In general, the 22 studies enrolled control patients with conditions that required medical examination or surgery.

#### 3.7.5. Medication

Ideally, patients should not receive medication that is expected to influence the levels of the investigated inflammatory markers [48]. However, only six of the 22 studies provided information regarding medication of patients [22, 24, 32, 38, 39, 41]. Four studies reported that patients received medication [22, 24, 38, 41], while two studies reported absence of medication [32, 39]. One study reported that patients with PHH secondarily to IVH received thrombolytic agents [41]. Another study reported that patients with PHH secondarily to SAH received a combination of nimopedine, a calcium channel blocker, and vasopressors [24].

#### 3.7.6. Preanalytical and Analytical Methods

*(1) CSF Sampling*. It remains controversial whether a protein concentration gradient exists from the ventricular to the lumbar space [49–53]. To rule out variations related to sample origin, CSF samples should originate from the same space [54]. Ten studies compared lumbar CSF from patients with hydrocephalus and control patients [25, 27, 31–33, 35, 37–39, 42], while eight studies compared ventricular CSF from patients with hydrocephalus to lumbar CSF from control patients [21, 23, 26, 28–30, 40, 41]. One study separately compared ventricular and lumbar CSF from patients with hydrocephalus to lumbar CSF from control patients [22], whereas another study mixed ventricular and lumbar CSF from a subset of patients with hydrocephalus [26]. A third study acquired ventricular and lumbar CSF from both patient groups and compared the ventricular and lumbar CSF samples separately [36]. Two studies did not adequately describe the origin of the CSF from one of the included patient groups [24, 34]. Two studies reported higher levels of inflammatory markers in lumbar CSF compared to ventricular CSF within patient groups [22, 36].

*(2) Preanalytical CSF Handling*. Preanalytical factors such as centrifugation, time from sampling to storage, storage material, and storage temperature are known to influence CSF analyses [55–58]. CSF samples are recommended to be centrifuged at 2000 × *g* for 10 min at 4°C or room temperature prior to storage at -80°C [55]. Fourteen studies centrifuged CSF samples, either prior to storage [21, 23–25, 28–30, 32, 33, 39] or after storage [31, 35, 37, 38]. Seven studies reported centrifugation speed and duration (2000-10,000 × *g*/2500-3000 rpm for 6-10 min) [28, 31–33, 35, 37, 38]. Four studies reported centrifugation temperature (4-7°C) [32, 33, 37, 38]. CSF samples are recommended to be stored within 1-2 hours of withdrawal to prevent variable results [55]. Eight studies reported that CSF samples were stored immediately [21–23, 26, 28–30, 32], while one study reported storage within 30 minutes [40]. The remaining studies did not specify the time from CSF sampling to storage. Polypropylene tubes are generally recommended for CSF storage as their low protein binding capacity minimizes the degree of adherence to tube walls [55–58]. However, only five studies provided information regarding the type of storage tubes used [25, 26, 37–39]. Two studies stored CSF in polypropylene tubes as recommended [25, 39]. Two studies used regular plastic tubes [37, 38]. The last study only reported use of cryogenic vials [26].

*(3) Selection of Inflammatory Markers for CSF Analysis*. The majority of the studies provided information in the introduction of their publication explaining why the inflammatory markers, or at least why some of them, were selected for analysis. Eight studies stated that some of the selected inflammatory markers had been detected previously in CSF from patients with hydrocephalus [23, 31, 33, 35–38, 41]. None of the studies stated that CSF was screened for presence of inflammatory markers in an unbiased manner, although one study stated that they “broadly investigated neuro-inflammatory processes” [35]. The study investigated 17 inflammatory markers, the highest number across all of the included studies [35]. Biased selection of inflammatory markers may hinder discovery of new markers that are worth investigating in relation to hydrocephalus development. Hence, as one study proposed, an unbiased exploration may be preferable in the future [22].

## 4. Discussion

In the current review, we systematically evaluated the existing literature on inflammatory markers in CSF from patients with hydrocephalus. We sought to identify promising inflammatory markers for future experimental research that aims to elucidate whether inflammatory markers can alter CSF dynamics and contribute to hydrocephalus development and progression. If neuroinflammation is involved in the pathogenesis of hydrocephalus, inflammatory markers may serve as novel disease biomarkers. Modulation of inflammatory pathways affects a range of systemic illnesses [59, 60] and may well serve as potential targets for pharmacological management of hydrocephalus. We reviewed the results presented in 22 studies, which investigated the presence of 58 inflammatory markers. Thirty-six inflammatory markers were investigated in iNPH patients, 37 were investigated in PHH patients, and 30 were investigated in patients with other hydrocephalus diagnoses. The inflammatory markers with similar CSF levels in hydrocephalus patients compared to control subjects were considered less promising, as they may not be relevant in the pathogenesis of hydrocephalus. For iNPH patients, IL-6, IL-1*β*, and LRG were associated with the most evidence of significantly increased CSF levels. For PHH patients, IL-6, IL-18, and VEGF were associated with the most evidence of significantly increased CSF levels. For patients with other hydrocephalus diagnoses, IFN-*γ* and VEGF were significantly increased in several types of hydrocephalus compared to controls.

### 4.1. Inflammatory Markers and Signaling Pathways in Hydrocephalus

Activation of the NF-*κ*B pathway was recently demonstrated to cause CSF hypersecretion and subsequent hydrocephalus in rats [15]. IL-6 dramatically rises in inflammatory conditions [61, 62], where IL-6 exerts chemotactic effects, stimulates production of acute-phase proteins, and regulates immune responses [63]. IL-6 has also been reported to activate the NF-*κ*B pathway [64]. IL-6 may therefore potentially promote choroidal CSF hypersecretion and hydrocephalus development by activating the NF-*κ*B pathway. IL-1*β* is secreted primarily by immune cells in situations of infection, cell injury, and inflammation [65]. Similarly to IL-6, the NF-*κ*B pathway can be initiated by IL-1*β* [66]. Hence, IL-1*β* also appears to have the prerequisites to promote choroidal CSF hypersecretion and cause hydrocephalus development.

LRG (leucine-rich *α*2-glycoprotein) is produced by various cell types including hepatocytes, astrocytes, neutrophils, and epithelial cells in the inflamed tissue. LRG levels rise in inflammatory conditions such as meningitis, tuberculosis, and rheumatoid arthritis and are therefore suggested to be a marker of inflammation similar to acute-phase proteins [25, 67–72]. LRG levels can be induced by inflammatory cytokines including IL-6 and IL-1*β* [67, 73]. Hence, the increased LRG levels in iNPH patients might be a secondary response to the increased IL-6 and IL-1*β* levels. LRG signals by binding to endoglin (ENG), a component of the receptor complex for TGF-*β*, and LRG is therefore proposed to modulate TGF-*β* signaling [74]. Although the TGF-*β* signaling pathway has been reported to crosstalk with the NF-*κ*B pathway [75], it is uncertain whether LRG, rather than TGF- *β*, would initiate such crosstalk and hence potentially promote choroidal CSF hypersecretion and hydrocephalus development. IL-18 is secreted by immune cells such as macrophages and nonimmune cells such as epithelial cells, keratinocytes, and osteoblasts [76]. IL-18 belongs to the same family of cytokines as IL-1*β* and therefore shares properties related to the receptor structure and signaling pathways, including activation of the NF-*κ*B pathway [77]. The increased IL-18 levels in PHH patients may therefore reflect presence of inflammation. VEGF serves a variety of roles during inflammation [78]. Amongst others, VEGF stimulates production of inflammatory cytokines [78]. Similarly to IL-6 and IL-1*β*, VEGF is capable of activating the NF-*κ*B pathway [79, 80], and its main receptor VEGFR2 is found on ependymal cells lining the ventricles [81]. In rats, intraventricular VEGF infusion has been reported to cause ventriculomegaly associated with disruption of the ventricular ependymal cell lining and loss of cilia, which have previously been associated with hydrocephalus development [82–84]. Hence, VEGF may promote hydrocephalus development and possibly contribute to disease progression by disrupting the normal CSF flow. IFN-*γ* is secreted primarily by immune cells [85, 86]. Once inflammation is initiated, IFN-*γ* intensifies the inflammatory response. The elevated IFN-*γ* levels in patients with other hydrocephalus diagnoses suggest presence of an ongoing inflammatory condition. Although IFN-*γ* primarily activates the JAK/STAT pathway [85], IFN-*γ* is also capable of activating the NF-*κ*B pathway [87]. Hence, IFN-*γ* has the prerequisites to promote choroidal CSF hypersecretion similarly to IL-6, IL-1*β*, IL-18, and VEGF.

The molecular mechanisms underlying choroidal CSF secretion are still investigated [88]. Except for malignancy-related CSF hypersecretion, activation of the NF-*κ*B pathway is the only mechanism currently demonstrated to promote choroidal CSF hypersecretion and cause development of hydrocephalus [15, 89, 90]. The ability of the inflammatory markers, IL-6, IL-1*β*, LRG, IL-18, VEGF, and IFN-*γ*, to promote choroidal CSF hypersecretion was therefore mainly discussed in relation to activation of the NF-*κ*B pathway. However, this does not exclude the possibility that activation of alternative, yet unidentified, pathways may promote choroidal CSF hypersecretion. Hence, future research may reveal additional molecular mechanisms involved in choroidal CSF secretion, which may be regulated by inflammatory markers. Inflammatory markers may also promote hydrocephalus development and contribute to disease progression in ways that do not involve choroidal CSF hypersecretion. Prolonged inflammation can lead to scarring and fibrosis [16, 17], which may disrupt the CSF flow and impair CSF reabsorption [7, 18].

### 4.2. Limitations

The systematic review included patients with hydrocephalus of various etiologies. Although the term “hydrocephalus” applies to all, the clinical presentations, radiological features, and etiologies vary between age groups. The classification of hydrocephalus is complex and ambiguous and thus an obvious challenge for a review of the present type, as the same clinical condition may be classified in more than one way—and the same classification can apply to different clinical conditions. In this review, we sought to overcome this by choosing etiologies as our defining parameter. Even though the results were separated into three main categories, patient heterogeneity within each category may have influenced the results. The category that comprised patients with other hydrocephalus diagnoses may especially have suffered from variations related to patient heterogeneity. Discrepancies in the diagnostic criteria combined with general lack of the stringent inclusion and exclusion criteria may also have contributed to patient heterogeneity. The control subjects were likewise heterogeneous in health status. The included studies had variations in sample sizes, and the majority of studies did not match patient groups on age and sex, which may also have influenced the results of the review. In addition, recruitment of patients from specialized clinics such as hydrocephalus units may have resulted in selection bias of patients with more pronounced cognitive and physiological impairments than encountered on average. The included studies analyzed CSF from children and adults, but the results were not separated accordingly. The inflammatory CSF profile of children and adults could possibly differ, and such information may thus have been lost. The category comprising PHH patients may have been particularly prone to variations related to age differences (preterm infants to adults). However, as some studies failed to report an age distribution or compared CSF from a mix of children and adults, results separated according to age would likewise have been associated with certain limitations.

Another limitation of this review is the heterogeneity in sampling and handling of CSF across the included studies. Several studies directly compare lumbar CSF with ventricular CSF which may have influenced the results due to differences in the protein content from the ventricles to the lumbar space. The influence of sample time on CSF protein concentration remains debatable [57]. However, only two studies reported that samples were collected at a specific time of day [38, 39]. The included studies also exhibited variation regarding the circumstances under which CSF samples were collected from patients with hydrocephalus. The variable circumstances of CSF sampling may raise concerns as to whether the CSF samples are compatible across the included studies. Hence, it cannot be excluded that the results presented in the review are affected by the heterogeneous circumstances of CSF sampling.

The review included inflammatory markers both directly and indirectly linked to inflammation. The definition of an indirect inflammatory marker falls within a grey area. Hence, some of the indirect inflammatory markers might be more relevant than others. The current review included indirect inflammatory markers that appeared relevant based on the identified studies in the original search. Regarding medication, it is surprising that only one study comment on the use of medication in relation to the investigated outcome [38]. It can be speculated whether some of the prescribed drugs such as prednisone can have influenced the results of the study [22]. However, since the majority of the included studies failed to report whether patients received medication, the bias introduced by medication cannot be properly assessed.

## 5. Conclusion

Despite the limitations, this systematic review presents a currently needed overview of the relevant literature and enables identification of promising inflammatory markers for future experimental research. In the current systematic review, we evaluated the existing literature on inflammatory markers in CSF from patients with hydrocephalus. We identified six inflammatory markers, IL-6, IL-1*β*, LRG, IL-18, VEGF, and IFN- *γ*, which were associated with the most evidence of increased levels in CSF from patients with hydrocephalus compared to control subjects. Future research should investigate whether these markers directly contribute to hydrocephalus development and progression. Such research will elucidate whether inflammatory markers may serve as novel disease biomarkers and whether their signaling pathways represent potential targets for pharmacological management of hydrocephalus. Future studies investigating the presence of inflammatory markers in CSF from patients with hydrocephalus should strive to eliminate the potential sources of bias presented in the current review. Efforts should be made to minimize patient heterogeneity. Patient diagnosis should occur in accordance with published international guidelines, and the inclusion and exclusion criteria should be clearly stated. Control subjects should, if possible, resemble healthy individuals. Studies should avoid small sample sizes, and patients should be matched on age and sex. CSF samples should preferably originate from the same space, be handled in the same manner, and if possible, be analyzed with the same analytical method. Furthermore, inflammatory markers should preferably be selected for analysis in an unbiased manner. The efforts done to eliminate the sources of bias may result in the identification of additional inflammatory markers, which are potentially involved in hydrocephalus development and progression.

## Figures and Tables

**Table 1:** Inflammatory markers in iNPH patients.

Result	Marker	References
↑↑	IL-6	[37, 38, 42]
	IL-1*β*	[37, 38]
	LRG	[33, 25]

**↑**	SP-G	[34]
	T*β*R-II	[33]

**↓**	TIMP-4	[26]

→ →	IL-8	[26, 36, 39]
	TNF-*α*	[36, 37, 42]
	CCL-2/MCP-1	[26, 36]
	IL-4	[37, 38]
	IL-33	[37, 38]
	sCD40L	[37, 38]

→	IL-12	[42]
	IL-12p40	[32]
	L-selectin	[26]
	MMP-9	[26]
	TGF-*β*2	[33]
	TIMP-1	[26]
	TIMP-2	[26]
	YKL-40	[27]

Contr	IL-21	[37, 38]
	TGF-*β*1	[26, 32, 33]
	IL-12p70	[32, 36]
	IL-17A	[37, 38]
	IL-22	[37, 38]
	IL-31	[37, 38]
	IL-10	[32, 36–39, 42]

↑↑ = most evidence: significantly increased in iNPH patients in two or more studies, and maximum one study reports a contradictory result. ↑ = limited evidence: significantly increased in iNPH patients in a single study. ↓ = limited evidence: significantly decreased in iNPH patients in a single study. → → = most evidence: no significant difference in two or more studies, and maximum one study reports a contradictory result. → = no significant difference in a single study. Contr = contradicting results. Abbreviations: IL: interleukin; LRG: leucine-rich *α*2-glycoprotein; SP-G: surfactant protein-G; T*β*R-II: transforming growth factor beta type II receptor; TIMP: tissue inhibitor of metalloproteinases; TNF: tumor necrosis factor; CCL: C-C motif chemokine ligand; MCP: monocyte chemoattractant protein; sCD40: soluble CD40 ligand; MMP: matrix metalloproteinase; TGF: transforming growth factor; YKL40: chitinase-3-like protein.

**Table 2:** Inflammatory markers in PHH patients.

Result	Marker	Reference
↑↑	IL-6 IL-18 VEGF	[26, 35] [29, 30] [21, 40]

↑	CCL-3/MIP-1*α* CCL-19 CXCL-10/IP-10 HGF HMGB1 IL-1*α* IL-4 IL-12 L1CAM MMP-9 FasR sFas SP-G sRAGE TGF-*β*2 TIMP-1	[35] [35] [35] [40] [28] [35] [35] [35] [31] [26] [40] [23] [34] [24] [41] [26]

↓	TIMP-4 XCL-1	[26] [35]

→ →	TGF-*β*1	[21, 35]

→	CXCL-11 CXCL-12 IL-10 L-selectin SCF FasL sFasL TIMP-2	[35] [35] [35] [26] [40] [40] [30] [26]

Contr	CCL-2/MCP-1 IL-1*β* IL-8 IFN-*γ* TNF-*α*	[26, 35] [29, 35] [26, 35] [30, 35] [26, 35, 40]

↑↑ = most evidence: significantly increased in PHH patients in two or more studies, and maximum one study reports a contradictory result. ↑ = limited evidence: significantly increased in PHH patients in a single study. ↓ = limited evidence: significantly decreased in PHH patients in a single study. → → = most evidence: no significant difference in two or more studies, and maximum one study reports a contradictory result. → = no significant difference in a single study. Contr = contradicting results. Abbreviations: IL: interleukin; VEGF: vascular endothelial growth factor; CCL: C-C motif chemokine ligand; MIP: macrophage inflammatory protein; CXCL: C-X-C motif chemokine ligand; IP: interferon gamma inducible protein; HGF: hepatocyte growth factor; HMGB1: high-mobility group box 1; L1CAM: L1 cell adhesion molecule; MMP: matrix metalloproteinase; FasR: Fas receptor; sFas: soluble Fas; SP-G: surfactant protein-G; sRAGE: soluble receptor for advanced glycation end products; TGF: transforming growth factor; TIMP: tissue inhibitor of metalloproteinases; XCL: X-C motif chemokine ligand; SCF: stem cell factor; FasL: Fas ligand; sFasL: soluble fas ligand; MCP: monocyte chemoattractant protein; IFN: interferon; TNF: tumor necrosis factor.

## Data Availability

All data is available on request.

## Supplementary Materials

Supplementary Materials Supplemental Table 1: overview of all inflammatory markers investigated in CSF from iNPH patients. Supplemental Table 2: overview of all inflammatory markers investigated in CSF from PHH patients. Supplemental Table 3: overview of all inflammatory markers investigated in CSF from patients with other hydrocephalus diagnoses.
