# Supplementary material for: Inflammatory Markers in Cerebrospinal Fluid from Patients with Hydrocephalus: A Systematic Literature Review
Source: Dis Markers. 2021 Feb 2;2021:8834822. doi: 10.1155/2021/8834822 (PMC7875647; doi:10.1155/2021/8834822)
Supplement: Supplementary Materials — Supplemental Table 1: overview of all inflammatory markers investigated in CSF from iNPH patients. Supplemental Table 2: overview of all inflammatory markers investigated in CSF from PHH patients. Supplemental Table 3: overview of all inflammatory markers investigated in CSF from patients with other hydrocephalus diagnoses. [file 8834822.f1.docx]

| **SUPPLEMENTARY TABLE 1** | | | | | | | | |
| --- | --- | --- | --- | --- | --- | --- | --- | --- |
| **Marker** | **Level** | **Patients** | **N** | **Age** | **Sex (M/F)** | **CSF** | **Method** | **Reference** |
| **CCL-2/MCP-1** | **↑** | iNPH  Ctrl | 28  20 | 69±7y  70±4y | 15/13  11/9 | L  L | ECL | [1] |
|  | → | iNPH  Ctrl | 10  5 | NR  NR | NR  NR | V  L | ELISA | [2] |
|  | → | iNPH  Ctrl | 48  23 | 73y  71y | 25/23  0/23 | L, V  L, V | Multiplex | [3] |
| **FGF-2** | ND | iNPH  Ctrl | 10  8 | NR  NR | NR  NR | V  L | ELISA | [2] |
| **IL-1β** | **↑** | iNPH  Ctrl | 20  20 | 65-80y  65-80y | NR  NR | L  L | Multiplex | [4] |
|  | **↑** | iNPH  Ctrl | 18  11 | 65-80y  NR | 11/7  3/8 | L  L | Multiplex | [5] |
|  | ND | iNPH  Ctrl | 48  23 | 73y  71y | 25/23  0/23 | L, V  L, V | Multiplex | [3] |
| **IL-2** | ND | iNPH  Ctrl | 48  23 | 73y  71y | 25/23  0/23 | L, V  L, V | Multiplex | [3] |
| **IL-4** | → | iNPH  Ctrl | 20  20 | 65-80y  65-80y | NR  NR | L  L | Multiplex | [4] |
|  | → | iNPH  Ctrl | 18  11 | 65-80y  NR | 11/7  3/8 | L  L | Multiplex | [5] |
|  | ND | iNPH  Ctrl | 48  23 | 73y  71y | 25/23  0/23 | L, V  L, V | Multiplex | [3] |
| **IL-5** | ND | iNPH  Ctrl | 48  23 | 73y  71y | 25/23  0/23 | L, V  L, V | Multiplex | [3] |
| **IL-6** | → | iNPH  Ctrl | 10  11 | NR  NR | NR  NR | V  L | ELISA | [2] |
|  | **↑** | iNPH  Ctrl | 20  20 | 65-80y  65-80y | NR  NR | L  L | Multiplex | [4] |
|  | **↑** | iNPH  Ctrl | 18  11 | 65-80y  NR | 11/7  3/8 | L  L | Multiplex | [5] |
|  | **↑** | iNPH  Ctrl | 5  2 | NR  NR | NR  NR | L  L | BD CBA kit | [6] |
| **IL-8** | → | iNPH  Ctrl | 28  20 | 69±7y  70±4y | 15/13  11/9 | L  L | ECL | [1] |
|  | → | iNPH  Ctrl | 10  11 | NR  NR | NR  NR | V  L | ELISA | [2] |
|  | **↑** | iNPH  Ctrl | 5  2 | NR  NR | NR  NR | L  L | BD CBA kit | [6] |
|  | → | iNPH  Ctrl | 48  23 | 73y  71y | 25/23  0/23 | L, V  L, V | Multiplex | [3] |
| **IL-10** | → | iNPH  Ctrl | 28  20 | 69±7y  70±4y | 15/13  11/9 | L  L | ECL | [1] |
|  | → | iNPH  Ctrl | 14  25 | 66y  69y | 9/5  15/10 | L  L | RIA | [7] |
|  | **↑** | iNPH  Ctrl | 20  20 | 65-80y  65-80y | NR  NR | L  L | Multiplex | [4] |
|  | **↑** | iNPH  Ctrl | 18  11 | 65-80y  NR | 11/7  3/8 | L  L | Multiplex | [5] |
|  | → | iNPH  Ctrl | 5  2 | NR  NR | NR  NR | L  L | BD CBA kit | [6] |
|  | ND | iNPH  Ctrl | 48  23 | 73y  71y | 25/23  0/23 | L, V  L, V | Multiplex | [3] |
| **IL-12** | → | iNPH  Ctrl | 5  2 | NR  NR | NR  NR | L  L | BD CBA kit | [6] |
| **IL-12 p40** | → | iNPH  Ctrl | 14  25 | 66y  69y | 9/5  15/10 | L  L | RIA | [7] |
| **IL-12 p70** | → | iNPH  Ctrl | 14  25 | 66y  69y | 9/5  15/10 | L  L | RIA | [7] |
|  | ND | iNPH  Ctrl | 48  23 | 73y  71y | 25/23  0/23 | L, V  L, V | Multiplex | [3] |
| **IL-13** | ND | iNPH  Ctrl | 48  23 | 73y  71y | 25/23  0/23 | L, V  L, V | Multiplex | [3] |
| **IL-17A** | → | iNPH  Ctrl | 20  20 | 65-80y  65-80y | NR  NR | L  L | Multiplex | [4] |
|  | ND | iNPH  Ctrl | 18  11 | 65-80y  NR | 11/7  3/8 | L  L | Multiplex | [5] |
| **IL-17F** | ND | iNPH  Ctrl | 20  20 | 65-80y  65-80y | NR  NR | L  L | Multiplex | [4] |
|  | ND | iNPH  Ctrl | 18  11 | 65-80y  NR | 11/7  3/8 | L  L | Multiplex | [5] |
| **IL-21** | → | iNPH  Ctrl | 20  20 | 65-80y  65-80y | NR  NR | L  L | Multiplex | [4] |
|  | **↑** | iNPH  Ctrl | 18  11 | 65-80y  NR | 11/7  3/8 | L  L | Multiplex | [5] |
| **IL-22** | → | iNPH  Ctrl | 20  20 | 65-80y  65-80y | NR  NR | L  L | Multiplex | [4] |
|  | ND | iNPH  Ctrl | 18  11 | 65-80y  NR | 11/7  3/8 | L  L | Multiplex | [5] |
| **IL-23** | ND | iNPH  Ctrl | 20  20 | 65-80y  65-80y | NR  NR | L  L | Multiplex | [4] |
|  | ND | iNPH  Ctrl | 18  11 | 65-80y  NR | 11/7  3/8 | L  L | Multiplex | [5] |
| **IL-25** | ND | iNPH  Ctrl | 20  20 | 65-80y  65-80y | NR  NR | L  L | Multiplex | [4] |
|  | ND | iNPH  Ctrl | 18  11 | 65-80y  NR | 11/7  3/8 | L  L | Multiplex | [5] |
| **IL-31** | → | iNPH  Ctrl | 20  20 | 65-80y  65-80y | NR  NR | L  L | Multiplex | [4] |
|  | ND | iNPH  Ctrl | 18  11 | 65-80y  NR | 11/7  3/8 | L  L | Multiplex | [5] |
| **IL-33** | → | iNPH  Ctrl | 20  20 | 65-80y  65-80y | NR  NR | L  L | Multiplex | [4] |
|  | → | iNPH  Ctrl | 18  11 | 65-80y  NR | 11/7  3/8 | L  L | Multiplex | [5] |
| **IFN-γ** | ND | iNPH  Ctrl | 14  25 | 66y  69y | 9/5  15/10 | L  L | RIA | [7] |
|  | ND | iNPH  Ctrl | 20  20 | 65-80y  65-80y | NR  NR | L  L | Multiplex | [4] |
|  | ND | iNPH  Ctrl | 18  11 | 65-80y  NR | 11/7  3/8 | L  L | Multiplex | [5] |
|  | ND | iNPH  Ctrl | 48  23 | 73y  71y | 25/23  0/23 | L, V  L, V | Multiplex | [3] |
| **LRG** | **↑** | iNPH  Ctrl | 21  14 | 72y  66y | 13/8  7/7 | L  L | ELISA | [8] |
|  | **↑** | iNPH  Ctrl | 100  26 | 76y  68y | 63/37  13/13 | L  L | ELISA | [9] |
| **L-selectin** | → | iNPH  Ctrl | 10  2 | NR  NR | NR  NR | V  L | ELISA | [2] |
| **MMP-9** | → | iNPH  Ctrl | 10  11 | NR  NR | NR  NR | V  L | ELISA | [2] |
| **sCD40L** | → | iNPH  Ctrl | 20  20 | 65-80y  65-80y | NR  NR | L  L | Multiplex | [4] |
|  | → | iNPH  Ctrl | 18  11 | 65-80y  NR | 11/7  3/8 | L  L | Multiplex | [5] |
| **SP-G** | **↑** | iNPH  Ctrl | 27  30 | 70y (31-84)  44y (0-84) | 18/9  15/15 | NR  L | ELISA | [10] |
| **TGF-β1** | ND | iNPH  Ctrl | 10  9 | NR  NR | NR  NR | V  L | ELISA | [2] |
|  | **↑** | iNPH  Ctrl | 21  14 | 72y  66y | 13/8  7/7 | L  L | ELISA | [8] |
|  | → | iNPH  Ctrl | 14  25 | 66y  69y | 9/5  15/10 | L  L | ELISA | [7] |
| **TGF-β2** | → | iNPH  Ctrl | 21  14 | 72y  66y | 13/8  7/7 | L  L | ELISA | [8] |
| **TGF-β3** | ND | iNPH  Ctrl | 21  14 | 72y  66y | 13/8  7/7 | L  L | ELISA | [8] |
| **TIMP-1** | → | iNPH  Ctrl | 10  10 | NR  NR | NR  NR | V  L | ELISA | [2] |
| **TIMP-2** | → | iNPH  Ctrl | 10  10 | NR  NR | NR  NR | V  L | ELISA | [2] |
| **TIMP-4** | ↓ | iNPH  Ctrl | 10  9 | NR  NR | NR  NR | V  L | ELISA | [2] |
| **TNF-α** | ND | iNPH  Ctrl | 10  3 | NR  NR | NR  NR | V  L | ELISA | [2] |
|  | → | iNPH  Ctrl | 20  20 | 65-80y  65-80y | NR  NR | L  L | Multiplex | [4] |
|  | **↑** | iNPH  Ctrl | 18  11 | 65-80y  NR | 11/7  3/8 | L  L | Multiplex | [5] |
|  | → | iNPH  Ctrl | 5  2 | NR  NR | NR  NR | L  L | BD CBA kit | [6] |
|  | → | iNPH  Ctrl | 48  23 | 73y  71y | 25/23  0/23 | L, V  L, V | Multiplex | [3] |
| **TβR-II** | **↑** | iNPH  Ctrl | 21  14 | 72y  66y | 13/8  7/7 | L  L | ELISA | [8] |
| **YKL-40** | → | iNPH  Ctrl | 20  20 | 71y  71y | 12/8  8/12 | L  L | ELISA | [11] |

**Supplementary Table 1:** **Inflammatory markers investigated in CSF from iNPH patients.**

Abbreviations: CCL = C-C motif chemokine ligand, FGF = fibroblast growth factor, IL = interleukin, IFN = interferon, LRG = Leucine-rich α2-glycoprotein, MCP = monocyte chemoattractant protein, MMP = matrix metalloproteinase, sCD40L = soluble CD40 ligand, SP-G = surfactant protein-G, TGF = transforming growth factor, TIMP = tissue inhibitor of metalloproteinases, TNF = tumor necrosis factor, TβR-II = transforming growth factor beta type II receptor, YKL40 = Chitinase-3-like protein 1, iNPH = idiopathic normal pressure hydrocephalus, Ctrl = controls, ND = not detected, NR = not reported, L = lumbar, V = ventricular, ECL = electrochemiluminescence, RIA= radioimmunoassay, ELISA = Enzyme-linked immunosorbent assay, BD CBA = BD cytometric bead assay, ↑ = increased levels vs. ctrl, → = levels not significantly different, ↓= decreased levels vs. ctrl. Age is reported as mean, mean ± standard deviation, mean (range), or range. Age is reported in years (y).

| **SUPPLEMENTARY TABLE 2** | | | | | | | | |
| --- | --- | --- | --- | --- | --- | --- | --- | --- |
| **Marker** | **Level** | **Patients** | **N** | **Age** | **Sex (M/F)** | **CSF** | **Method** | **Study** |
| **CCL-2/MCP-1** | → | PHH  Ctrl | 14  31 | 26±2w  30±3w | 9/5  24/7 | L  L | Multiplex | [12] |
|  | **↑** | PHH  Ctrl | 7  5 | NR  NR | NR  NR | Mix  L | ELISA | [2] |
| **CCL-3/MIP-1α** | **↑** | PHH  Ctrl | 14  31 | 26±2w  30±3w | 9/5  24/7 | L  L | ELISA | [12] |
| **CCL-19** | **↑** | PHH  Ctrl | 14  31 | 26±2w  30±3w | 9/5  24/7 | L  L | Multiplex | [12] |
| **CXCL-10/IP-10** | **↑** | PHH  Ctrl | 14  31 | 26±2w  30±3w | 9/5  24/7 | L  L | Multiplex | [12] |
| **CXCL-11** | → | PHH  Ctrl | 14  31 | 26±2w  30±3w | 9/5  24/7 | L  L | Multiplex | [12] |
| **CXCL-12** | → | PHH  Ctrl | 14  31 | 26±2w  30±3w | 9/5  24/7 | L  L | ELISA | [12] |
| **FasL** | → | PHH  Ctrl | 6  7 | 86d  38d | 2/4  2/5 | V  L | Microarray | [13] |
| **FasR** | **↑** | PHH  Ctrl | 6  7 | 86d  38d | 2/4  2/5 | V  L | Microarray | [13] |
| **FGF-2** | ND | PHH  Ctrl | 13  8 | NR  NR | NR  NR | Mix  L | ELISA | [2] |
| **HGF** | **↑** | PHH  Ctrl | 6  7 | 86d  38d | 2/4  2/5 | V  L | Microarray | [13] |
| **HMGB1** | **↑** | PHH  Ctrl | 10  8 | 61y  NR | 6/4  NR | V  L | ELISA | [14] |
| **IGF-1** | ND | PHH  Ctrl | 6  7 | 86d  38d | 2/4  2/5 | V  L | Microarray | [13] |
| **IL-1α** | **↑** | PHH  Ctrl | 14  31 | 26±2w  30±3w | 9/5  24/7 | L  L | Multiplex | [12] |
| **IL-1β** | → | PHH  Ctrl | 14  31 | 26±2w  30±3w | 9/5  24/7 | L  L | Multiplex | [12] |
|  | **↑** | PHH  Ctrl | 27  20 | 27w+1d  29w+2d | NR  NR | V  L | ELISA | [15] |
| **IL-4** | **↑** | PHH  Ctrl | 14  31 | 26±2w  30±3w | 9/5  24/7 | L  L | Multiplex | [12] |
| **IL-6** | **↑** | PHH  Ctrl | 14  31 | 26±2w  30±3w | 9/5  24/7 | L  L | Multiplex | [12] |
|  | **↑** | PHH  Ctrl | 11  11 | NR  NR | NR  NR | Mix  L | ELISA | [2] |
|  | → | PHH  Ctrl | 6  7 | 86d  38d | 2/4  2/5 | V  L | Microarray | [13] |
| **IL-8** | → | PHH  Ctrl | 14  31 | 26±2w  30±3w | 9/5  24/7 | L  L | Multiplex | [12] |
|  | **↑** | PHH  Ctrl | 13  11 | NR  NR | NR  NR | Mix  L | ELISA | [2] |
| **IL-10** | → | PHH  Ctrl | 14  31 | 26±2w  30±3w | 9/5  24/7 | L  L | Multiplex | [12] |
| **IL-12** | **↑** | PHH  Ctrl | 14  31 | 26±2w  30±3w | 9/5  24/7 | L  L | Multiplex | [12] |
| **IL-18** | **↑** | PHH  Ctrl | 27  20 | 27w+1d  29w+2d | NR  NR | V  L | ELISA | [15] |
|  | **↑** | PHH  Ctrl | 6  15 | 27-54w  24-54w | NR  NR | V  L | ELISA | [16] |
| **IFN-γ** | → | PHH  Ctrl | 14  31 | 26±2w  30±3w | 9/5  24/7 | L  L | Multiplex | [12] |
|  | **↑** | PHH  Ctrl | 6  15 | 27-54w  24-54w | NR  NR | V  L | ELISA | [16] |
| **L1CAM** | **↑** | PHH  Ctrl | 12  33 | 27w  30w | NR  NR | L  L | ELISA | [17] |
| **L-selectin** | → | PHH  Ctrl | 7  2 | NR  NR | NR  NR | Mix  L | ELISA | [2] |
| **MMP-9** | **↑** | PHH  Ctrl | 12  11 | NR  NR | NR  NR | Mix  L | ELISA | [2] |
| **SCF** | → | PHH  Ctrl | 6  7 | 86d  38d | 2/4  2/5 | V  L | Microarray | [13] |
| **sFas** | **↑** | PHH  Ctrl | 29  24 | 26w+5d  32w+6d | NR  NR | V  L | ELISA | [18] |
| **sFasL** | → | PHH  Ctrl | 6  15 | 27-54w  24-54w | NR  NR | V  L | ELISA | [16] |
| **SP-G** | **↑** | PHH  Ctrl | 12  30 | 60y (17-82)  44y (0-84) | 7/5  15/15 | NR  L | ELISA | [10] |
| **sRAGE** | **↑** | PHH  Ctrl | 20  20 | 58y  NR | NR  NR | V  NR | ELISA | [19] |
| **TGF-β1** | → | PHH  Ctrl | 14  31 | 26±2w  30±3w | 9/5  24/7 | L  L | ELISA | [12] |
|  | → | PHH  Ctrl | 18  11 | 25w+0d  33w+4d | NR  NR | V  L | ELISA | [20] |
|  | ND | PHH  Ctrl | 14  9 | NR  NR | NR  NR | Mix  L | ELISA | [2] |
|  | **↑** | PHH  Ctrl | 10  12 | 23-37w  1-4w | NR  NR | V  L | Quantikine IA | [21] |
| **TGF-β2** | **↑** | PHH  Ctrl | 10  12 | 23-37w  1-4w | NR  NR | V  L | Quantikine IA | [21] |
| **TIMP-1** | **↑** | PHH  Ctrl | 13  10 | NR  NR | NR  NR | Mix  L | ELISA | [2] |
| **TIMP-2** | → | PHH  Ctrl | 13  10 | NR  NR | NR  NR | Mix  L | ELISA | [2] |
| **TIMP-4** | **↓** | PHH  Ctrl | 12  9 | NR  NR | NR  NR | Mix  L | ELISA | [2] |
| **TNF-α** | **↑** | PHH  Ctrl | 14  31 | 26±2w  30±3w | 9/5  24/7 | L  L | Multiplex | [12] |
|  | ND | PHH  Ctrl | 10  3 | NR  NR | NR  NR | Mix  L | ELISA | [2] |
|  | → | PHH  Ctrl | 6  7 | 86d  38d | 2/4  2/5 | V  L | Microarray | [13] |
| **VEGF** | **↑** | PHH  Ctrl | 18  11 | 25w+0d  33w+4d | NR  NR | V  L | ELISA | [20] |
|  | **↑** | PHH  Ctrl | 6  7 | 86d  38d | 2/4  2/5 | V  L | Microarray | [13] |
| **XCL-1** | **↓** | PHH  Ctrl | 14  31 | 26±2w  30±3w | 9/5  24/7 | L  L | ELISA | [12] |

**Supplementary Table 2:** **Inflammatory markers investigated in CSF from PHH patients**.

Abbreviations: CCL = C-C motif chemokine ligand, MCP = monocyte chemoattractant protein, MIP = macrophage inflammatory protein, CXCL = C-X-C motif chemokine ligand, IP = interferon gamma inducible protein, FasL = Fas ligand, FasR = Fas receptor, FGF = fibroblast growth factor, HGF = hepatocyte growth factor, HMGB1 = high-mobility group box 1, IGF = insulin growth factor, IL = interleukin, IFN = interferon, L1CAM = L1 cell adhesion molecule, MMP = matrix metalloproteinase, SCF = stem cell factor, sFas = soluble Fas, sFasL = soluble fas ligand, SP-G = surfactant protein-G, sRAGE = soluble receptor for advanced glycation end products, TGF = transforming growth factor, TIMP = tissue inhibitor of metalloproteinases, TNF = tumor necrosis factor, VEGF = vascular endothelial growth factor, XCL = X-C motif chemokine ligand, PHH = posthhemorrhagic hydrocephalus, Ctrl = controls, ND = not detected, NR = not reported, ↑ = increased levels vs. ctrl, → = levels not significantly different, ↓ = decreased levels vs. ctrl, L = lumbar, V = ventricular, ELISA = Enzyme-linked immunosorbent assay, IA = immunoassay. Note: the term PHH covers both patients with acute and chronic hydrocephalus. Age is reported as mean, mean ± standard deviation, mean (range), or range. Age is reported in years (y), weeks (w), or days (d).

| **SUPPLEMENTARY TABLE 3** | | | | | | | | |
| --- | --- | --- | --- | --- | --- | --- | --- | --- |
| **Marker** | **Level** | **Patients** | **N** | **Age** | **Sex (M/F)** | **CSF** | **Method** | **Study** |
| **CCL-2/MCP1** | → | HUA  Ctrl | 6  5 | NR  NR | NR  NR | V  L | ELISA | [2] |
|  | **↑** | HTBM  Ctrl | 44  11 | 3y  3y | 28/16  8/3 | L,V  L | Milliplex | [22] |
| **CCL-3/MIP-1α** | **↑** | HTBM  Ctrl | 44  11 | 3y  3y | 28/16  8/3 | L,V  L | Milliplex | [22] |
| **CXCL-10/IP-10** | **↑** | HTBM  Ctrl | 44  11 | 3y  3y | 28/16  8/3 | L,V  L | Milliplex | [22] |
| **FasL** | → | FOH  Ctrl | 7  7 | 56d  38d | 5/2  2/5 | V  L | Microarray | [13] |
|  | → | LOH  Ctrl | 7  7 | 95d  38d | 6/1  2/5 | V  L | Microarray | [13] |
|  | → | SBH  Ctrl | 8  7 | 90d  38d | 1/7  2/5 | V  L | Microarray | [13] |
| **FasR** | → | FOH  Ctrl | 7  7 | 56d  38d | 5/2  2/5 | V  L | Microarray | [13] |
|  | → | LOH  Ctrl | 7  7 | 95d  38d | 6/1  2/5 | V  L | Microarray | [13] |
|  | **↑** | SBH  Ctrl | 8  7 | 90d  38d | 1/7  2/5 | V  L | Microarray | [13] |
| **FGF-2** | ND | HUA  Ctrl | 6  8 | NR  NR | NR  NR | V  L | ELISA | [2] |
| **GRO** | **↑** | HTBM  Ctrl | 44  11 | 3y  3y | 28/16  8/3 | L,V  L | Milliplex | [22] |
| **HGF** | → | FOH  Ctrl | 7  7 | 56d  38d | 5/2  2/5 | V  L | Microarray | [13] |
|  | → | LOH  Ctrl | 7  7 | 95d  38d | 6/1  2/5 | V  L | Microarray | [13] |
|  | → | SBH  Ctrl | 8  7 | 90d  38d | 1/7  2/5 | V  L | Microarray | [13] |
| **IGF-1** | ND | FOH  Ctrl | 7  7 | 56d  38d | 5/2  2/5 | V  L | Microarray | [13] |
|  | ND | LOH  Ctrl | 7  7 | 95d  38d | 6/1  2/5 | V  L | Microarray | [13] |
|  | ND | SBH  Ctrl | 8  7 | 90d  38d | 1/7  2/5 | V  L | Microarray | [13] |
| **IL-1β** | **↑** | HTBM  Ctrl | 44  11 | 3y  3y | 28/16  8/3 | L,V  L | Milliplex | [22] |
| **IL-1Ra** | **↑** | HTBM  Ctrl | 44  11 | 3y  3y | 28/16  8/3 | L,V  L | Milliplex | [22] |
| **IL-6** | → | AH  Ctrl | 6  2 | NR  NR | NR  NR | L  L | BD CBA kit | [6] |
|  | → | HBA  Ctrl | 5  2 | NR  NR | NR  NR | L  L | BD CBA kit | [6] |
|  | **↑** | HUA  Ctrl | 8  11 | NR  NR | NR  NR | V  L | ELISA | [2] |
|  | → | FOH  Ctrl | 7  7 | 56d  38d | 5/2  2/5 | V  L | Microarray | [13] |
|  | → | LOH  Ctrl | 7  7 | 95d  38d | 6/1  2/5 | V  L | Microarray | [13] |
|  | → | SBH  Ctrl | 8  7 | 90d  38d | 1/7  2/5 | V  L | Microarray | [13] |
|  | **↑** | HTBM  Ctrl | 44  11 | 3y  3y | 28/16  8/3 | L,V  L | Milliplex | [22] |
| **IL-8** | → | AH  Ctrl | 6  2 | NR  NR | NR  NR | L  L | BD CBA kit | [6] |
|  | **↑** | HBA  Ctrl | 5  2 | NR  NR | NR  NR | L  L | BD CBA kit | [6] |
|  | → | HUA  Ctrl | 8  11 | NR  NR | NR  NR | V  L | ELISA | [2] |
|  | **↑** | HTBM  Ctrl | 44  11 | 3y  3y | 28/16  8/3 | L,V  L | Milliplex | [22] |
| **IL-10** | → | AH  Ctrl | 6  2 | NR  NR | NR  NR | L  L | BD CBA kit | [6] |
|  | → | HBA  Ctrl | 5  2 | NR  NR | NR  NR | L  L | BD CBA kit | [6] |
|  | **↑** | HTBM  Ctrl | 44  11 | 3y  3y | 28/16  8/3 | L,V  L | Milliplex | [22] |
| **IL-12** | → | AH  Ctrl | 6  2 | NR  NR | NR  NR | L  L | BD CBA kit | [6] |
|  | → | HBA  Ctrl | 5  2 | NR  NR | NR  NR | L  L | BD CBA kit | [6] |
| **IL-12p40** | **↑** | HTBM  Ctrl | 44  11 | 3y  3y | 28/16  8/3 | L,V  L | Milliplex | [22] |
| **IL-18** | **↑** | SBH  Ctrl | 20  15 | 27-54w  24-54w | NR  NR | V  L | ELISA | [16] |
|  | **↑** | ASH  Ctrl | 4  15 | 27-54w  24-54w | NR  NR | V  L | ELISA | [16] |
| **IFN-γ** | **↑** | HTBM  Ctrl | 44  11 | 3y  3y | 28/16  8/3 | L,V  L | Milliplex | [22] |
|  | **↑** | SBH  Ctrl | 20  15 | 27-54w  24-54w | NR  NR | V  L | ELISA | [16] |
|  | **↑** | ASH  Ctrl | 4  15 | 27-54w  24-54w | NR  NR | V  L | ELISA | [16] |
| **L-selectin** | → | HUA  Ctrl | 5  2 | NR  NR | NR  NR | V  L | ELISA | [2] |
| **MMP-9** | → | HUA  Ctrl | 7  11 | NR  NR | NR  NR | V  L | ELISA | [2] |
| **RANTES** | → | HTBM  Ctrl | 44  11 | 3y  3y | 28/16  8/3 | L,V  L | Milliplex | [22] |
| **SCF** | → | FOH  Ctrl | 7  7 | 56d  38d | 5/2  2/5 | V  L | Microarray | [13] |
|  | → | LOH  Ctrl | 7  7 | 95d  38d | 6/1  2/5 | V  L | Microarray | [13] |
|  | **↑** | SBH  Ctrl | 8  7 | 90d  38d | 1/7  2/5 | V  L | Microarray | [13] |
| **sFasL** | → | SBH  Ctrl | 20  15 | 27-54w  24-54w | NR  NR | V  L | ELISA | [16] |
|  | → | ASH  Ctrl | 4  15 | 27-54w  24-54w | NR  NR | V  L | ELISA | [16] |
| **SP-G** | → | ASH  Ctrl | 21  30 | 19y (0-65)  44y (0-84) | 8/13  15/15 | NR  L | ELISA | [10] |
|  | → | AcH  Ctrl | 12  30 | 15y (0-75)  44y (0-84) | 5/7  15/15 | NR  L | ELISA | [10] |
| **TGF-β1** | ND | HUA  Ctrl | 7  9 | NR  NR | NR  NR | V  L | ELISA | [2] |
|  | → | CH  Ctrl | 9  11 | 34w+3d  33w+4d | NR  NR | V  L | ELISA | [20] |
| **TIMP-1** | → | HUA  Ctrl | 6  10 | NR  NR | NR  NR | V  L | ELISA | [2] |
| **TIMP-2** | **↓** | HUA  Ctrl | 6  10 | NR  NR | NR  NR | V  L | ELISA | [2] |
| **TIMP-4** | **↓** | HUA  Ctrl | 7  9 | NR  NR | NR  NR | V  L | ELISA | [2] |
| **TNF-α** | → | AH  Ctrl | 6  2 | NR  NR | NR  NR | L  L | BD CBA kit | [6] |
|  | → | HBA  Ctrl | 5  2 | NR  NR | NR  NR | L  L | BD CBA kit | [6] |
|  | ND | HUA  Ctrl | 4  3 | NR  NR | NR  NR | V  L | ELISA | [2] |
|  | → | FOH  Ctrl | 7  7 | 56d  38d | 5/2  2/5 | V  L | Microarray | [13] |
|  | → | LOH  Ctrl | 7  7 | 95d  38d | 6/1  2/5 | V  L | Microarray | [13] |
|  | → | SBH  Ctrl | 8  7 | 90d  38d | 1/7  2/5 | V  L | Microarray | [13] |
|  | **↑** | HTBM  Ctrl | 44  11 | 3y  3y | 28/16  8/3 | L,V  L | Milliplex | [22] |
| **VEGF** | **↑** | CH  Ctrl | 9  11 | 34w+3d  33w+4d | NR  NR | V  L | ELISA | [20] |
|  | → | FOH  Ctrl | 7  7 | 56d  38d | 5/2  2/5 | V  L | Microarray | [13] |
|  | → | LOH  Ctrl | 7  7 | 95d  38d | 6/1  2/5 | V  L | Microarray | [13] |
|  | **↑** | SBH  Ctrl | 8  7 | 90d  38d | 1/7  2/5 | V  L | Microarray | [13] |
|  | **↑** | HTBM  Ctrl | 44  11 | 3y  3y | 28/16  8/3 | L,V  L | Milliplex | [22] |

**Supplementary Table 3:** **Inflammatory markers investigated in CSF from patients with other hydrocephalus diagnoses.** Abbreviations: CCL = C-C motif chemokine ligand, MCP = monocyte chemoattractant protein, MIP = macrophage inflammatory protein, CXCL = C-X-C motif chemokine ligand, IP = interferon gamma inducible protein, FasL = Fas ligand, FasR = Fas receptor, FGF = fibroblast growth factor, GRO = growth regulated oncogene, HGF = hepatocyte growth factor, IGF = insulin growth factor, IL = interleukin, IFN = interferon, MMP = matrix metalloproteinase, RANTES = normal T-cell expressed and secreted protein, SCF = stem cell factor, sFasL = soluble fas ligand, SP-G = surfactant protein-G, TGF = transforming growth factor, TIMP = tissue inhibitor of metalloproteinases, TNF = tumor necrosis factor, VEGF = vascular endothelial growth factor, Ctrl = controls, ND = not detected, NR = not reported, ↑ = increased levels vs. ctrl, → = levels not significantly different, ↓ = decreased levels vs. ctrl, L = lumbar, V = ventricular, ELISA = Enzyme-linked immunosorbent assay, IA = immunoassay, AH = arrested hydrocephalus. HBA = hydrocephalus with brain atrophy, CH = congenital hydrocephalus (mix of different subtypes), HUA = hydrocephalus following the embolization of unruptured intracranial aneurysms, FOH = fetal onset hydrocephalus, LOH = late onset hydrocephalus, SBH = spina bifida hydrocephalus, HTBM = hydrocephalus associated with tuberculous meningitis, ASH = aqueduct stenosis hydrocephalus. AcH = acute hydrocephalus, Age is reported as mean, mean ± standard deviation, mean (range), or range. Age is reported in years (y), weeks (w), or days (d).

# **REFERENCES – Supplementary tables**

[1] A. Jeppsson, H. Zetterberg, K. Blennow, and C. Wikkelso, “Idiopathic normal-pressure hydrocephalus: Pathophysiology and diagnosis by CSF biomarkers,” *Neurology*, vol. 80, no. 15, pp. 1385–1392, Apr. 2013.

[2] M. Killer *et al.*, “Cytokine and Growth Factor Concentration in Cerebrospinal Fluid from Patients with Hydrocephalus Following Endovascular Embolization of Unruptured Aneurysms in Comparison with Other Types of Hydrocephalus,” *Neurochem. Res.*, vol. 35, no. 10, pp. 1652–1658, Oct. 2010.

[3] O. T. Pyykkö *et al.*, “Cerebrospinal Fluid Biomarker and Brain Biopsy Findings in Idiopathic Normal Pressure Hydrocephalus,” *PLoS One*, vol. 9, no. 3, p. e91974, Mar. 2014.

[4] L. Sosvorova, J. Vcelak, M. Mohapl, J. Vitku, M. Bicikova, and R. Hampl, “Selected pro- and anti-inflammatory cytokines in cerebrospinal fluid in normal pressure hydrocephalus,” *Neuro Endocrinol Lett*, vol. 35, no. 7, pp. 586–593, 2014.

[5] L. Sosvorova *et al.*, “The comparison of selected cerebrospinal fluid and serum cytokine levels in patients with multiple sclerosis and normal pressure hydrocephalus.,” *Neuro Endocrinol. Lett.*, vol. 36, no. 6, pp. 564–71, Dec. 2015.

[6] K. Czubowicz, M. Głowacki, E. Fersten, E. Kozłowska, R. P. Strosznajder, and Z. Czernicki, “Levels of selected pro- and anti-inflammatory cytokines in cerebrospinal fluid in patients with hydrocephalus,” *Folia Neuropathol.*, vol. 55, no. 4, pp. 301–307, 2017.

[7] E. Rota, G. Bellone, P. Rocca, B. Bergamasco, G. Emanuelli, and P. Ferrero, “Increased intrathecal TGF-β1, but not IL-12, IFN-γ and IL-10 levels in Alzheimer’s disease patients,” *Neurol. Sci.*, vol. 27, no. 1, pp. 33–39, Apr. 2006.

[8] X. Li, M. Miyajima, C. Jiang, and H. Arai, “Expression of TGF-βs and TGF-β type II receptor in cerebrospinal fluid of patients with idiopathic normal pressure hydrocephalus,” *Neurosci. Lett.*, vol. 413, no. 2, pp. 141–144, Feb. 2007.

[9] M. Miyajima *et al.*, “Leucine-Rich α2-Glycoprotein Is a Novel Biomarker of Neurodegenerative Disease in Human Cerebrospinal Fluid and Causes Neurodegeneration in Mouse Cerebral Cortex,” *PLoS One*, vol. 8, no. 9, p. e74453, Sep. 2013.

[10] M. Krause *et al.*, “Localization, Occurrence, and CSF Changes of SP-G, a New Surface Active Protein with Assumable Immunoregulatory Functions in the CNS,” *Mol. Neurobiol.*, vol. 56, no. 4, pp. 2433–2439, Apr. 2019.

[11] A. Jeppsson, M. Höltta, H. Zetterberg, K. Blennow, C. Wikkelsø, and M. Tullberg, “Amyloid mis-metabolism in idiopathic normal pressure hydrocephalus,” *Fluids Barriers CNS*, vol. 13, no. 1, p. 13, Dec. 2016.

[12] G. Habiyaremye *et al.*, “Chemokine and cytokine levels in the lumbar cerebrospinal fluid of preterm infants with post-hemorrhagic hydrocephalus,” *Fluids Barriers CNS*, vol. 14, no. 1, p. 35, Dec. 2017.

[13] I. Naureen *et al.*, “Fingerprint changes in CSF composition associated with different aetiologies in human neonatal hydrocephalus: inflammatory cytokines,” *Child’s Nerv. Syst.*, vol. 30, no. 7, pp. 1155–1164, Jul. 2014.

[14] B. Sokół *et al.*, “HMGB1 Level in Cerebrospinal Fluid as a Marker of Treatment Outcome in Patients with Acute Hydrocephalus Following Aneurysmal Subarachnoid Hemorrhage,” *J. Stroke Cerebrovasc. Dis.*, vol. 24, no. 8, pp. 1897–1904, Aug. 2015.

[15] T. Schmitz *et al.*, “Interleukin-1β, Interleukin-18, and Interferon-γ Expression in the Cerebrospinal Fluid of Premature Infants with Posthemorrhagic Hydrocephalus—Markers of White Matter Damage?,” *Pediatr. Res.*, vol. 61, no. 6, pp. 722–726, Jun. 2007.

[16] D. A. Sival, U. Felderhoff-Müser, T. Schmitz, E. W. Hoving, C. Schaller, and A. Heep, “Neonatal high pressure hydrocephalus is associated with elevation of pro-inflammatory cytokines IL-18 and IFNγ in cerebrospinal fluid,” *Cerebrospinal Fluid Res.*, vol. 5, no. 1, p. 21, Dec. 2008.

[17] D. M. Morales *et al.*, “Lumbar Cerebrospinal Fluid Biomarkers of Posthemorrhagic Hydrocephalus of Prematurity,” *Neurosurgery*, vol. 80, no. 1, p. 1, Aug. 2016.

[18] T. Schmitz, U. Felderhoff-Mueser, M. Sifringer, F. Groenendaal, S. Kampmann, and A. Heep, “Expression of soluble Fas in the cerebrospinal fluid of preterm infants with posthemorrhagic hydrocephalus and cystic white matter damage,” *J. Perinat. Med.*, vol. 39, no. 1, pp. 83–88, Jan. 2011.

[19] B. Sokół *et al.*, “Increase of Soluble RAGE in Cerebrospinal Fluid following Subarachnoid Haemorrhage,” *Biomed Res. Int.*, vol. 2017, pp. 1–7, 2017.

[20] A. Heep *et al.*, “Vascular Endothelial Growth Factor and Transforming Growth Factor-β1 Are Highly Expressed in the Cerebrospinal Fluid of Premature Infants with Posthemorrhagic Hydrocephalus,” *Pediatr. Res.*, vol. 56, no. 5, pp. 768–774, Nov. 2004.

[21] A. Whitelaw, S. Christie, and I. Pople, “Transforming Growth Factor-β1: A Possible Signal Molecule for Posthemorrhagic Hydrocephalus?,” *Pediatr. Res.*, vol. 46, no. 5, pp. 576–576, Nov. 1999.

[22] U. K. Rohlwink *et al.*, “Biomarkers of Cerebral Injury and Inflammation in Pediatric Tuberculous Meningitis,” *Clin. Infect. Dis.*, vol. 65, no. 8, pp. 1298–1307, Oct. 2017.
